# A general passion

**DOI:** 10.1371/journal.pgen.1011291

**Published:** 2024-05-30

**Authors:** Gregory S. Barsh, Gregory P. Copenhaver

**Affiliations:** 1 HudsonAlpha Institute for Biotechnology, Huntsville, Alabama, United States of America; 2 Department of Genetics, Stanford University School of Medicine, Stanford, California, United States of America; 3 Department of Biology, University of North Carolina at Chapel Hill, Chapel Hill, North Carolina, United States of America; 4 Integrative Program for Biological and Genome Sciences, University of North Carolina at Chapel Hill, Chapel Hill, North Carolina, United States of America; Pacific Northwest Research Institute, UNITED STATES

Aristotle, Avicenna (Ibn Sina), Benjamin Franklin, Émilie du Châtelet, Ada Lovelace, and Jagdish Chandra Bose were all great scientists—indeed, greater than we (the authors) could possibly aspire to be. Nonetheless, we feel a certain kinship with them around a shared passion, the love of the pursuit of knowledge across a broad spectrum. Scientists today operate with an unprecedented wealth of tools, data, and technology, but the cost of fully leveraging those resources often means committing to deep specialization within a niche in your field. We’re all familiar with these kinds of conversations when meeting another scientist:

Scientist 1: *Hi*, *nice to meet you*, *I’m a [insert field]ist*.

Scientist 2: *Nice to meet you*, *I’m also a [insert field]ist*. *What do you work on*?

Scientist 1: *Oh*, *I study process X*, *in organism Y*, *primarily using Z tools*.

Scientist 2: *Ah*, *that’s why we haven’t met before*, *I work in organism W*.

The benefit of that kind of deep specialization at an individual level is being able to focus on, if not master, a narrow band of subject matter in an increasingly complex information and technological environment. At a broader level, it has resulted in remarkably rapid progress in some fields—witness the astonishingly fast recent development of the mRNA-based vaccines. But unlike the polymaths listed above, fewer and fewer of us have the latitude or encouragement to apply our curiosity and creativity to adjacent or even entirely different fields.

For the last decade, *PLOS Genetics* has given us the latitude and encouragement to be generalists. As co-Editors in Chief for *PLOS Genetics* and as section editors for the General Genetics section, one of us has learned more about plant genetics and chromosome biology, one of us has learned more about medical genetics and cancer biology, and, together, we have learned more about all areas of genetics. We are grateful to *PLOS Genetics* for pushing us out of our comfortable bubbles; we are especially grateful to the scientists across a broad array of disciplines who have submitted their work to be considered by our journal.

Naturally, our roles as Editors-in-Chief have been much more than a class in general genetics. Thinking about science communication from different perspectives has given us a new appreciation for the challenges and opportunities in science publishing, from open data access to publication ethics to how peer review should work. All three of those areas have their share of challenges, but one area that does not is our engagement with a diverse group of associate editors in evolutionary, microbial, plant, human, and theoretical genetics as well as epigenetics, methods, and computational biology. That aspect of the journey has been more rewarding than either of us could have imagined when we began. We have grown as scientists, gained perspectives that were previously obscure for us, and, most importantly, it has introduced us to a community of friends and colleagues we would otherwise have been unlikely to connect with.

The time has come for us to hand the Editor-in-Chief reigns to a new set of leaders who will bring their own wisdom and fresh ideas to the journal to ensure that it remains as vibrant and exciting as ever. We expect and, indeed hope, that the next Editors-in-Chief will move *PLOS Genetics* in new directions, and we look forward to following, supporting, and contributing to those efforts. At the same time, we urge the next Editors-in-Chief to remain committed to core principles established when *PLOS Genetics* was founded. As a publishing company, PLOS has seen several generations of leaders, strategic plans, and organizational structures—in our opinion, PLOS today is more mature than it was when we started. But what makes *PLOS Genetics* unique, and what we have advocated and, on several occasions, argued for during our tenure, is that the mantra, “by working scientists, for working scientists,” is more than just a catchphrase. Editorial decisions should be made solely by working scientists and not professional editors. The structure, organization, and policies of the journal should be a collaboration in which working scientists and not professional editors play the leading role. Staying true to these principles will ensure that *PLOS Genetics* remains, truly, a community journal.

**Fig 1:**
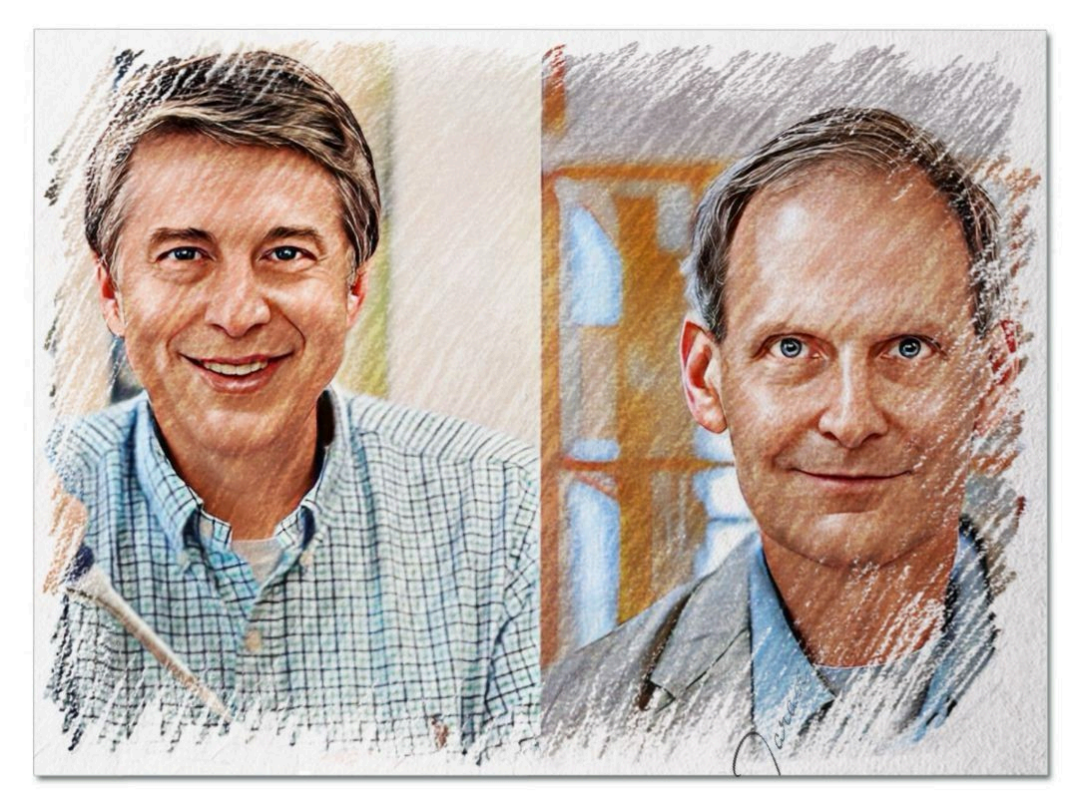
Gregory P. Copenhaver (left) and Gregory S. Barsh (right).

